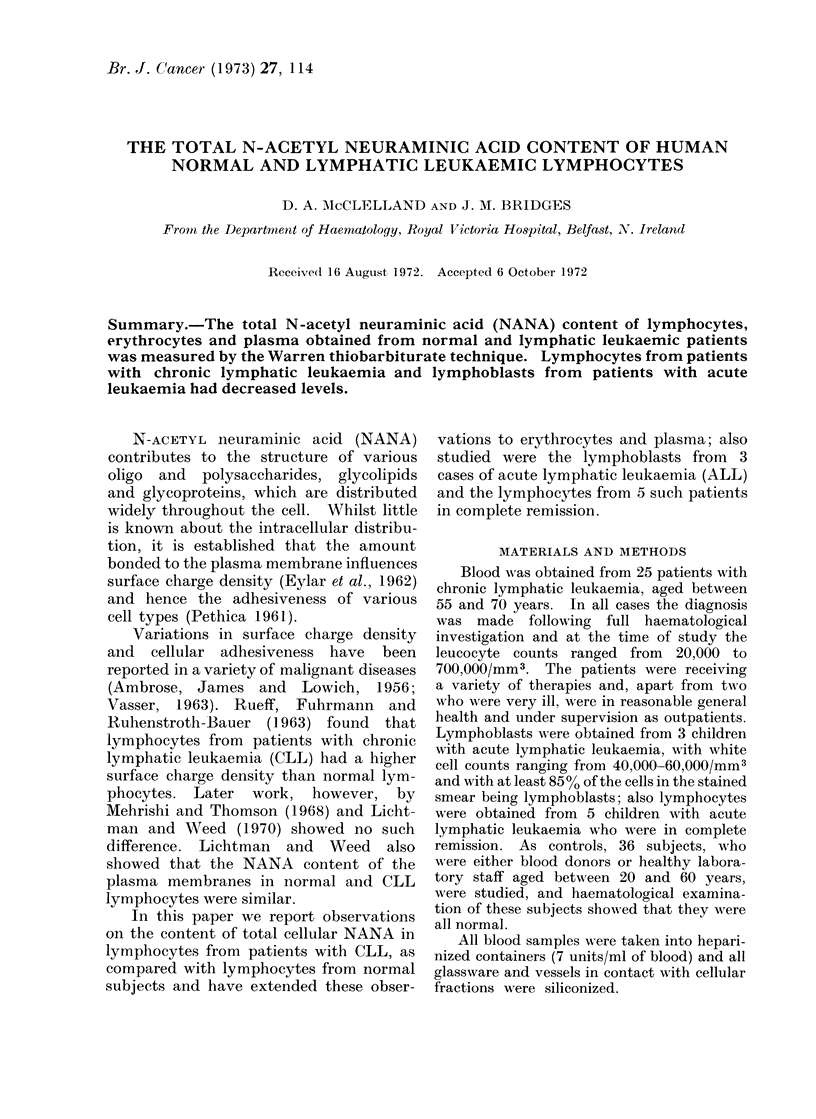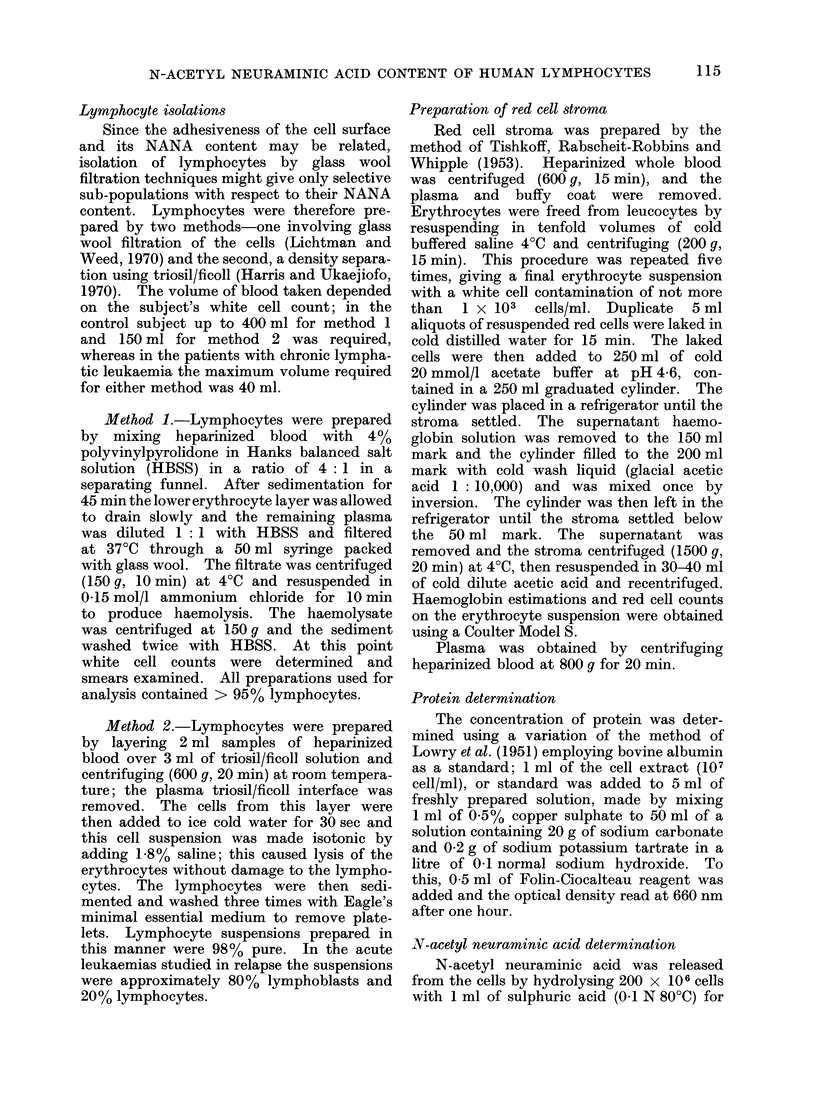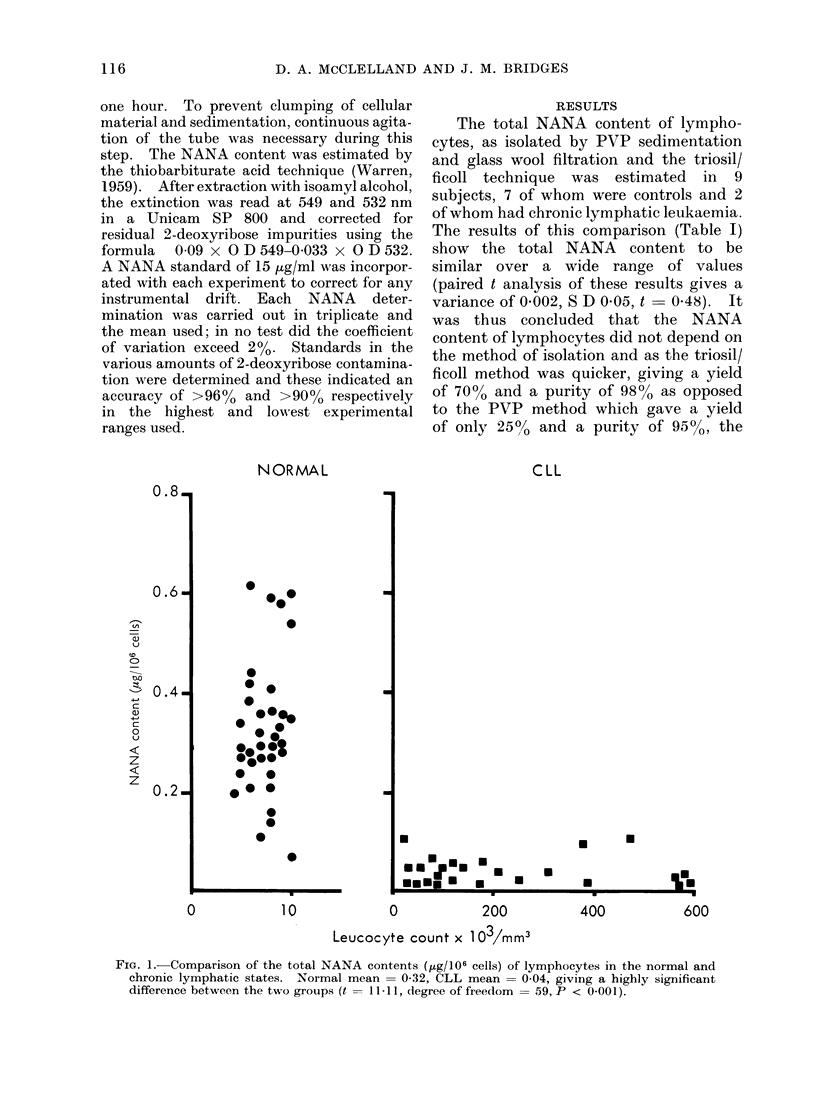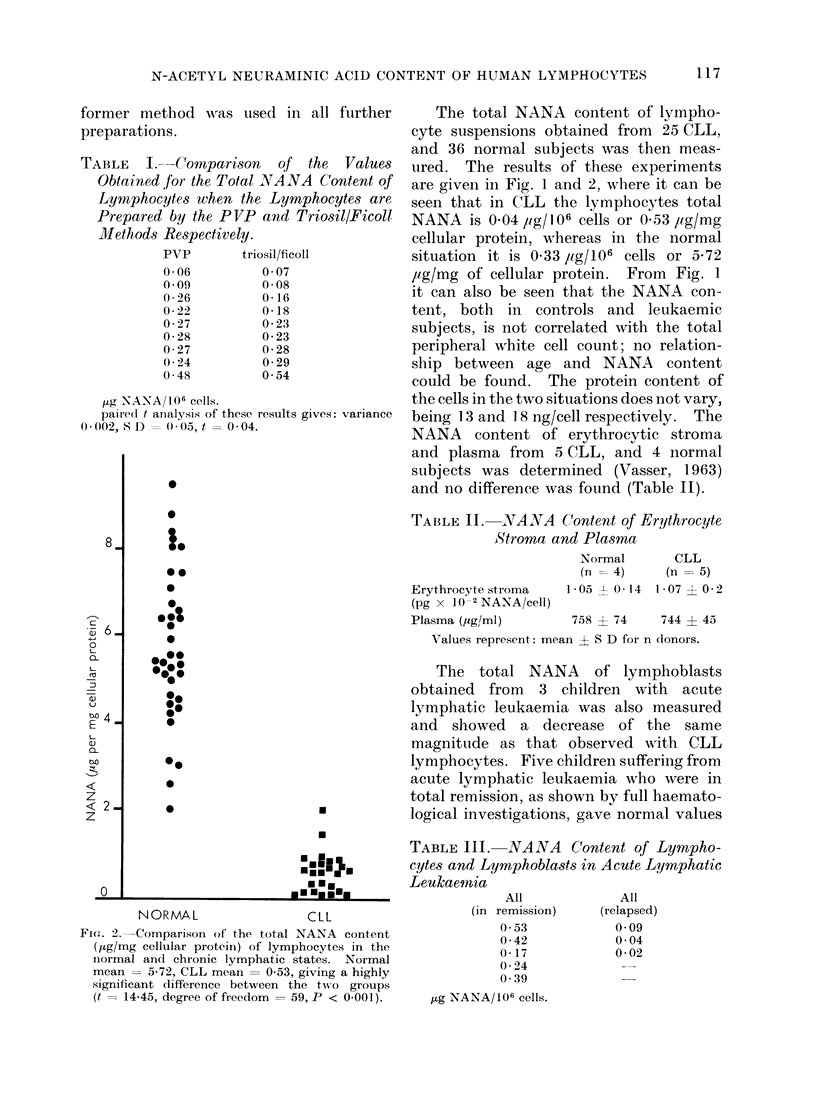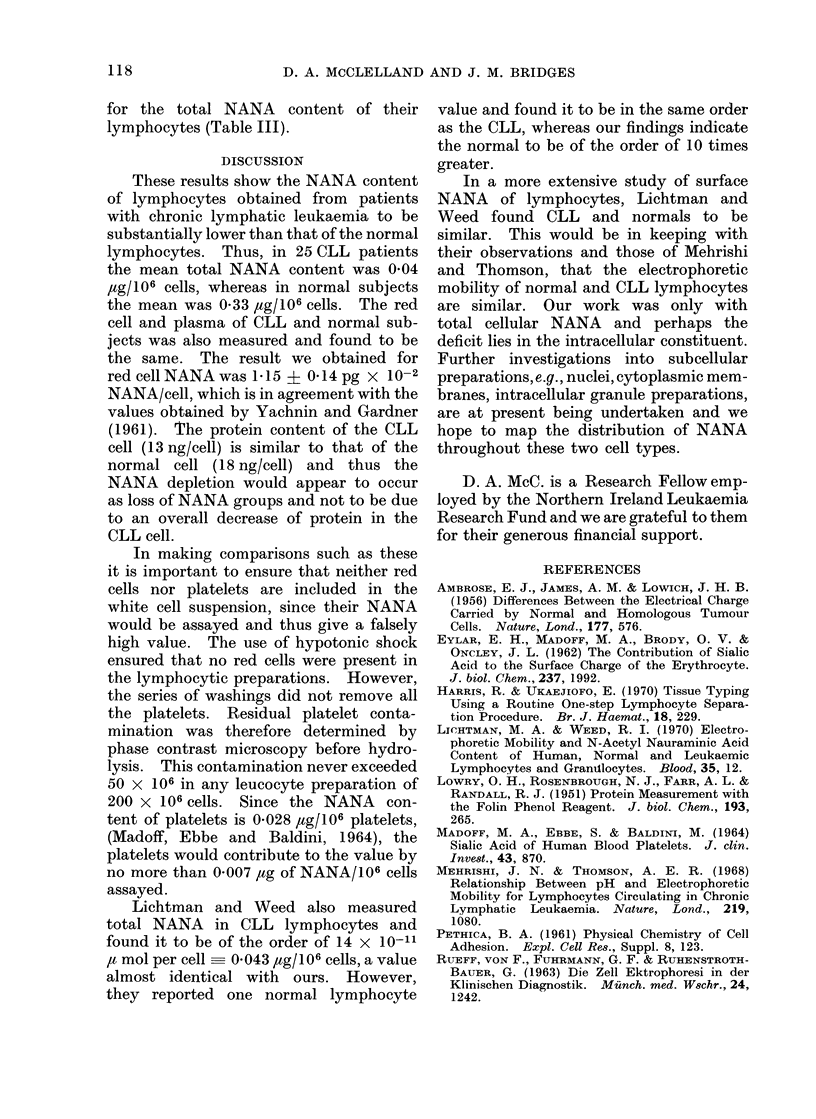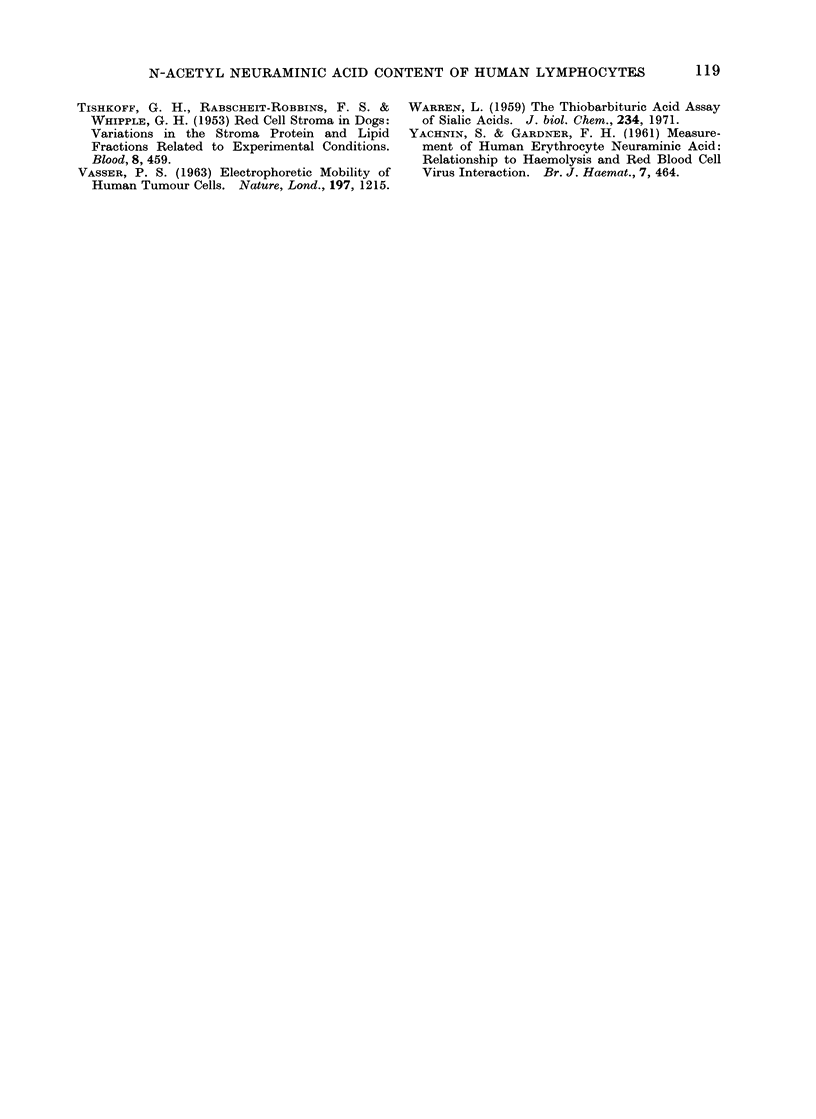# The Total N-Acetyl Neuraminic Acid Content of Human Normal and Lymphatic Leukaemic Lymphocytes

**DOI:** 10.1038/bjc.1973.15

**Published:** 1973-02

**Authors:** D. A. McClelland, J. M. Bridges

## Abstract

The total N-acetyl neuraminic acid (NANA) content of lymphocytes, erythrocytes and plasma obtained from normal and lymphatic leukaemic patients was measured by the Warren thiobarbiturate technique. Lymphocytes from patients with chronic lymphatic leukaemia and lymphoblasts from patients with acute leukaemia had decreased levels.


*Br. J. Cancer* (1973) **27**, 114

### THE TOTAL N-ACETYL NEURAMINIC ACID CONTENT OF HUMAN NORMAL AND LYMPHATIC LEUKAEMIC LYMPHOCYTES

D. A. McCLELLAND AND J. M. BRIDGES

*From the Department of Haematology, Royal Victoria Hospital, Belfast, N. Ireland*

Received 16 August 1972. Accepted 6 October 1972

**Summary.—The total N-acetyl neuraminic acid (NANA) content of lymphocytes, erythrocytes and plasma obtained from normal and lymphatic leukaemic patients was measured by the Warren thiobarbiturate technique. Lymphocytes from patients with chronic lymphatic leukaemia and lymphoblasts from patients with acute leukaemia had decreased levels.**

N-ACETYL neuraminic acid (NANA) contributes to the structure of various oligo and polysaccharides, glycolipids and glycoproteins, which are distributed widely throughout the cell. Whilst little is known about the intracellular distribution, it is established that the amount bonded to the plasma membrane influences surface charge density (Eylar *et al.*, 1962) and hence the adhesiveness of various cell types (Pethica 1961).

Variations in surface charge density and cellular adhesiveness have been reported in a variety of malignant diseases (Ambrose, James and Lowich, 1956; Vasser, 1963). Rueff, Fuhrmann and Ruhenstroth-Bauer (1963) found that lymphocytes from patients with chronic lymphatic leukaemia (CLL) had a higher surface charge density than normal lymphocytes. Later work, however, by Mehrishi and Thomson (1968) and Lichtman and Weed (1970) showed no such difference. Lichtman and Weed also showed that the NANA content of the plasma membranes in normal and CLL lymphocytes were similar.

In this paper we report observations on the content of total cellular NANA in lymphocytes from patients with CLL, as compared with lymphocytes from normal subjects and have extended these observations to erythrocytes and plasma; also studied were the lymphoblasts from 3 cases of acute lymphatic leukaemia (ALL) and the lymphocytes from 5 such patients in complete remission.

### MATERIALS AND METHODS

Blood was obtained from 25 patients with chronic lymphatic leukaemia, aged between 55 and 70 years. In all cases the diagnosis was made following full haematological investigation and at the time of study the leucocyte counts ranged from 20,000 to 700,000/mm$^3$. The patients were receiving a variety of therapies and, apart from two who were very ill, were in reasonable general health and under supervision as outpatients. Lymphoblasts were obtained from 3 children with acute lymphatic leukaemia, with white cell counts ranging from 40,000–60,000/mm$^3$ and with at least 85% of the cells in the stained smear being lymphoblasts; also lymphocytes were obtained from 5 children with acute lymphatic leukaemia who were in complete remission. As controls, 36 subjects, who were either blood donors or healthy laboratory staff aged between 20 and 60 years, were studied, and haematological examination of these subjects showed that they were all normal.

All blood samples were taken into heparinized containers (7 units/ml of blood) and all glassware and vessels in contact with cellular fractions were siliconized.

*Lymphocyte isolations*

Since the adhesiveness of the cell surface and its NANA content may be related, isolation of lymphocytes by glass wool filtration techniques might give only selective sub-populations with respect to their NANA content. Lymphocytes were therefore prepared by two methods—one involving glass wool filtration of the cells (Lichtman and Weed, 1970) and the second, a density separation using triosil/ficoll (Harris and Ukaejiofo, 1970). The volume of blood taken depended on the subject's white cell count; in the control subject up to 400 ml for method 1 and 150 ml for method 2 was required, whereas in the patients with chronic lymphatic leukaemia the maximum volume required for either method was 40 ml.

*Method 1.*—Lymphocytes were prepared by mixing heparinized blood with 4% polyvinylpyrolidone in Hanks balanced salt solution (HBSS) in a ratio of 4 : 1 in a separating funnel. After sedimentation for 45 min the lower erythrocyte layer was allowed to drain slowly and the remaining plasma was diluted 1 : 1 with HBSS and filtered at 37°C through a 50 ml syringe packed with glass wool. The filtrate was centrifuged (150 *g*, 10 min) at 4°C and resuspended in 0·15 mol/l ammonium chloride for 10 min to produce haemolysis. The haemolysate was centrifuged at 150 *g* and the sediment washed twice with HBSS. At this point white cell counts were determined and smears examined. All preparations used for analysis contained > 95% lymphocytes.

*Method 2.*—Lymphocytes were prepared by layering 2 ml samples of heparinized blood over 3 ml of triosil/ficoll solution and centrifuging (600 *g*, 20 min) at room temperature; the plasma triosil/ficoll interface was removed. The cells from this layer were then added to ice cold water for 30 sec and this cell suspension was made isotonic by adding 1·8% saline; this caused lysis of the erythrocytes without damage to the lymphocytes. The lymphocytes were then sedimented and washed three times with Eagle's minimal essential medium to remove platelets. Lymphocyte suspensions prepared in this manner were 98% pure. In the acute leukaemias studied in relapse the suspensions were approximately 80% lymphoblasts and 20% lymphocytes.

*Preparation of red cell stroma*

Red cell stroma was prepared by the method of Tishkoff, Rabscheit-Robbins and Whipple (1953). Heparinized whole blood was centrifuged (600 *g*, 15 min), and the plasma and buffy coat were removed. Erythrocytes were freed from leucocytes by resuspending in tenfold volumes of cold buffered saline 4°C and centrifuging (200 *g*, 15 min). This procedure was repeated five times, giving a final erythrocyte suspension with a white cell contamination of not more than $1 \times 10^3$ cells/ml. Duplicate 5 ml aliquots of resuspended red cells were laked in cold distilled water for 15 min. The laked cells were then added to 250 ml of cold 20 mmol/l acetate buffer at pH 4·6, contained in a 250 ml graduated cylinder. The cylinder was placed in a refrigerator until the stroma settled. The supernatant haemoglobin solution was removed to the 150 ml mark and the cylinder filled to the 200 ml mark with cold wash liquid (glacial acetic acid 1 : 10,000) and was mixed once by inversion. The cylinder was then left in the refrigerator until the stroma settled below the 50 ml mark. The supernatant was removed and the stroma centrifuged (1500 *g*, 20 min) at 4°C, then resuspended in 30–40 ml of cold dilute acetic acid and recentrifuged. Haemoglobin estimations and red cell counts on the erythrocyte suspension were obtained using a Coulter Model S.

Plasma was obtained by centrifuging heparinized blood at 800 *g* for 20 min.

*Protein determination*

The concentration of protein was determined using a variation of the method of Lowry *et al.* (1951) employing bovine albumin as a standard; 1 ml of the cell extract ($10^7$ cell/ml), or standard was added to 5 ml of freshly prepared solution, made by mixing 1 ml of 0·5% copper sulphate to 50 ml of a solution containing 20 g of sodium carbonate and 0·2 g of sodium potassium tartrate in a litre of 0·1 normal sodium hydroxide. To this, 0·5 ml of Folin-Ciocalteau reagent was added and the optical density read at 660 nm after one hour.

*N-acetyl neuraminic acid determination*

N-acetyl neuraminic acid was released from the cells by hydrolysing $200 \times 10^6$ cells with 1 ml of sulphuric acid (0·1 N 80°C) for one hour. To prevent clumping of cellular material and sedimentation, continuous agitation of the tube was necessary during this step. The NANA content was estimated by the thiobarbiturate acid technique (Warren, 1959). After extraction with isoamyl alcohol, the extinction was read at 549 and 532 nm in a Unicam SP 800 and corrected for residual 2-deoxyribose impurities using the formula 0·09 × O D 549–0·033 × O D 532. A NANA standard of 15 μg/ml was incorporated with each experiment to correct for any instrumental drift. Each NANA determination was carried out in triplicate and the mean used; in no test did the coefficient of variation exceed 2%. Standards in the various amounts of 2-deoxyribose contamination were determined and these indicated an accuracy of >96% and >90% respectively in the highest and lowest experimental ranges used.

### RESULTS

The total NANA content of lymphocytes, as isolated by PVP sedimentation and glass wool filtration and the triosil/ficoll technique was estimated in 9 subjects, 7 of whom were controls and 2 of whom had chronic lymphatic leukaemia. The results of this comparison (Table I) show the total NANA content to be similar over a wide range of values (paired $t$ analysis of these results gives a variance of 0·002, S D 0·05, $t = 0{\cdot}48$). It was thus concluded that the NANA content of lymphocytes did not depend on the method of isolation and as the triosil/ficoll method was quicker, giving a yield of 70% and a purity of 98% as opposed to the PVP method which gave a yield of only 25% and a purity of 95%, the

FIG. 1.—Comparison of the total NANA contents (μg/$10^6$ cells) of lymphocytes in the normal and chronic lymphatic states. Normal mean = 0·32, CLL mean = 0·04, giving a highly significant difference between the two groups ($t$ = 11·11, degree of freedom = 59, $P$ < 0·001).

former method was used in all further preparations.

TABLE I.—*Comparison of the Values Obtained for the Total NANA Content of Lymphocytes when the Lymphocytes are Prepared by the PVP and Triosil/Ficoll Methods Respectively.*

| PVP | triosil/ficoll |
|---|---|
| 0·06 | 0·07 |
| 0·09 | 0·08 |
| 0·26 | 0·16 |
| 0·22 | 0·18 |
| 0·27 | 0·23 |
| 0·28 | 0·23 |
| 0·27 | 0·28 |
| 0·24 | 0·29 |
| 0·48 | 0·54 |

μg NANA/$10^6$ cells.
paired *t* analysis of these results gives: variance 0·002, S D = 0·05, *t* = 0·04.

FIG. 2.—Comparison of the total NANA content (μg/mg cellular protein) of lymphocytes in the normal and chronic lymphatic states. Normal mean = 5·72, CLL mean = 0·53, giving a highly significant difference between the two groups (*t* = 14·45, degree of freedom = 59, $P < 0{\cdot}001$).

The total NANA content of lymphocyte suspensions obtained from 25 CLL, and 36 normal subjects was then measured. The results of these experiments are given in Fig. 1 and 2, where it can be seen that in CLL the lymphocytes total NANA is 0·04 μg/$10^6$ cells or 0·53 μg/mg cellular protein, whereas in the normal situation it is 0·33 μg/$10^6$ cells or 5·72 μg/mg of cellular protein. From Fig. 1 it can also be seen that the NANA content, both in controls and leukaemic subjects, is not correlated with the total peripheral white cell count; no relationship between age and NANA content could be found. The protein content of the cells in the two situations does not vary, being 13 and 18 ng/cell respectively. The NANA content of erythrocytic stroma and plasma from 5 CLL, and 4 normal subjects was determined (Vasser, 1963) and no difference was found (Table II).

TABLE II.—*NANA Content of Erythrocyte Stroma and Plasma*

| | Normal (n = 4) | CLL (n = 5) |
|---|---|---|
| Erythrocyte stroma (pg × $10^{-2}$ NANA/cell) | 1·05 ± 0·14 | 1·07 ± 0·2 |
| Plasma (μg/ml) | 758 ± 74 | 744 ± 45 |

Values represent: mean ± S D for n donors.

The total NANA of lymphoblasts obtained from 3 children with acute lymphatic leukaemia was also measured and showed a decrease of the same magnitude as that observed with CLL lymphocytes. Five children suffering from acute lymphatic leukaemia who were in total remission, as shown by full haematological investigations, gave normal values

TABLE III.—*NANA Content of Lymphocytes and Lymphoblasts in Acute Lymphatic Leukaemia*

| All (in remission) | All (relapsed) |
|---|---|
| 0·53 | 0·09 |
| 0·42 | 0·04 |
| 0·17 | 0·02 |
| 0·24 | — |
| 0·39 | — |

μg NANA/$10^6$ cells.

for the total NANA content of their lymphocytes (Table III).

### DISCUSSION

These results show the NANA content of lymphocytes obtained from patients with chronic lymphatic leukaemia to be substantially lower than that of the normal lymphocytes. Thus, in 25 CLL patients the mean total NANA content was 0·04 $\mu g/10^6$ cells, whereas in normal subjects the mean was 0·33 $\mu g/10^6$ cells. The red cell and plasma of CLL and normal subjects was also measured and found to be the same. The result we obtained for red cell NANA was $1{\cdot}15 \pm 0{\cdot}14$ pg $\times\ 10^{-2}$ NANA/cell, which is in agreement with the values obtained by Yachnin and Gardner (1961). The protein content of the CLL cell (13 ng/cell) is similar to that of the normal cell (18 ng/cell) and thus the NANA depletion would appear to occur as loss of NANA groups and not to be due to an overall decrease of protein in the CLL cell.

In making comparisons such as these it is important to ensure that neither red cells nor platelets are included in the white cell suspension, since their NANA would be assayed and thus give a falsely high value. The use of hypotonic shock ensured that no red cells were present in the lymphocytic preparations. However, the series of washings did not remove all the platelets. Residual platelet contamination was therefore determined by phase contrast microscopy before hydrolysis. This contamination never exceeded $50 \times 10^6$ in any leucocyte preparation of $200 \times 10^6$ cells. Since the NANA content of platelets is 0·028 $\mu g/10^6$ platelets, (Madoff, Ebbe and Baldini, 1964), the platelets would contribute to the value by no more than 0·007 $\mu g$ of NANA/$10^6$ cells assayed.

Lichtman and Weed also measured total NANA in CLL lymphocytes and found it to be of the order of $14 \times 10^{-11}$ $\mu$ mol per cell $\equiv 0{\cdot}043\ \mu g/10^6$ cells, a value almost identical with ours. However, they reported one normal lymphocyte value and found it to be in the same order as the CLL, whereas our findings indicate the normal to be of the order of 10 times greater.

In a more extensive study of surface NANA of lymphocytes, Lichtman and Weed found CLL and normals to be similar. This would be in keeping with their observations and those of Mehrishi and Thomson, that the electrophoretic mobility of normal and CLL lymphocytes are similar. Our work was only with total cellular NANA and perhaps the deficit lies in the intracellular constituent. Further investigations into subcellular preparations, *e.g.*, nuclei, cytoplasmic membranes, intracellular granule preparations, are at present being undertaken and we hope to map the distribution of NANA throughout these two cell types.

D. A. McC. is a Research Fellow employed by the Northern Ireland Leukaemia Research Fund and we are grateful to them for their generous financial support.

### REFERENCES

AMBROSE, E. J., JAMES, A. M. & LOWICH, J. H. B. (1956) Differences Between the Electrical Charge Carried by Normal and Homologous Tumour Cells. *Nature, Lond.*, **177**, 576.

EYLAR, E. H., MADOFF, M. A., BRODY, O. V. & ONCLEY, J. L. (1962) The Contribution of Sialic Acid to the Surface Charge of the Erythrocyte. *J. biol. Chem.*, **237**, 1992.

HARRIS, R. & UKAEJIOFO, E. (1970) Tissue Typing Using a Routine One-step Lymphocyte Separation Procedure. *Br. J. Haemat.*, **18**, 229.

LICHTMAN, M. A. & WEED, R. I. (1970) Electrophoretic Mobility and N-Acetyl Nauraminic Acid Content of Human, Normal and Leukaemic Lymphocytes and Granulocytes. *Blood*, **35**, 12.

LOWRY, O. H., ROSENBROUGH, N. J., FARR, A. L. & RANDALL, R. J. (1951) Protein Measurement with the Folin Phenol Reagent. *J. biol. Chem.*, **193**, 265.

MADOFF, M. A., EBBE, S. & BALDINI, M. (1964) Sialic Acid of Human Blood Platelets. *J. clin. Invest.*, **43**, 870.

MEHRISHI, J. N. & THOMSON, A. E. R. (1968) Relationship Between pH and Electrophoretic Mobility for Lymphocytes Circulating in Chronic Lymphatic Leukaemia. *Nature, Lond.*, **219**, 1080.

PETHICA, B. A. (1961) Physical Chemistry of Cell Adhesion. *Expl. Cell Res.*, Suppl. 8, 123.

RUEFF, VON F., FUHRMANN, G. F. & RUHENSTROTH-BAUER, G. (1963) Die Zell Ektrophoresi in der Klinischen Diagnostik. *Münch. med. Wschr.*, **24**, 1242.

TISHKOFF, G. H., RABSCHEIT-ROBBINS, F. S. & WHIPPLE, G. H. (1953) Red Cell Stroma in Dogs: Variations in the Stroma Protein and Lipid Fractions Related to Experimental Conditions. *Blood*, **8**, 459.

VASSER, P. S. (1963) Electrophoretic Mobility of Human Tumour Cells. *Nature, Lond.*, **197**, 1215.

WARREN, L. (1959) The Thiobarbituric Acid Assay of Sialic Acids. *J. biol. Chem.*, **234**, 1971.

YACHNIN, S. & GARDNER, F. H. (1961) Measurement of Human Erythrocyte Neuraminic Acid: Relationship to Haemolysis and Red Blood Cell Virus Interaction. *Br. J. Haemat.*, **7**, 464.